# Two genera of Braconinae (Hymenoptera, Braconidae) in China, with descriptions of four new species

**DOI:** 10.3897/zookeys.61.450

**Published:** 2010-10-11

**Authors:** Yi-Ping Wang, Xue-Xin Chen, Hong Wu, Jun-Hua He

**Affiliations:** Institute of Insect Science, College of Agriculture and Biotechnology, Zhejiang University, Hangzhou 310029, China; 1 Institute of Insect Science, College of Agriculture and Biotechnology, Zhejiang University, Hangzhou 310029, China; 2 College of Forestry and Biotechnology, Zhejiang Forestry University, Lin’an 311300, China

**Keywords:** Braconidae, Braconinae, Dolabraulax, Scutibracon, new species, China

## Abstract

Two genera, namely Dolabraulax Quicke and Scutibracon Quicke of Braconinae (Hymenoptera: Braconidae) from China are studied for the first time, and four new species, namely Dolabraulax jigongshanus Wang & Chen, **sp. n.**, Dolabraulax flavus Wang & Chen, **sp. n.**, Dolabraulax brevivena Wang & Chen, **sp. n.** and Scutibracon  fujianensis Wang & Chen, sp. n. are fully described and illustrated. The examined specimens are deposited in the Parasitic Hymenoptera Collection, Zhejiang University, Hangzhou, China (ZJUH).

## Introduction

The Braconinae is one of the largest and the most diverse cosmopolitansubfamiliesof Braconidae with about 2900 described species of about 180 genera worldwide, and mainly occurring in tropical and subtropical regions but particularly rich in the Indo-Australian and Afrotropical regions (Yu et al. 2005). The vast majority of species are ectoparasitoids principally of coleopterous and lepidopterous hosts although a few attack Diptera, Hymenoptera-Symphyta and possibly Homoptera, and one group, Aspidobraconina, are endoparasitic on pupa. Some species may be effective biocontrol agents to suppress agro-forestry insect pest populations (Quicke and Ingram 1993).

China is among the most diverse regions for braconids in the world because of large variation in climate, and its vast area, but unfortunately its fauna is poorly known. This is part of our on-going study of the subfamily Braconinae (Wang et al. 2003a, 2003b, 2003c, 2003d, 2004, 2006a, 2006b, 2006c, 2006d, 2007, 2008, 2009a, 2009b). The present paper deals with two genera, namely Dolabraulax Quicke and Scutibracon Quicke of Braconinae from China for the first time, and four new species, i.e. Dolabraulax jigongshanus Wang & Chen, sp. n., Dolabraulax flavus Wang & Chen, sp. n., Dolabraulax brevivena Wang & Chen, sp. n. and Scutibracon fujianensis Wang & Chen, sp. n. are recognized, which are fully described and illustrated. The examined specimens are deposited in the Parasitic Hymenoptera Collection, Zhejiang University, Hangzhou, China (ZJUH).

The morphological terminology used in this paper follows that of van Achterberg (1979), Harris (1979) and Quicke (1987). All descriptions and measurements were made under a Leica MZ 12.5 stereomicroscope (Wetzlar, Germany), and photos taken by a digital camera (Q-Imaging, Micropublisher 3.3 RTV) attached to a Leica MZ APO stereomicroscope (Wetzlar, Germany) using Synoptics Auto-Montage version 5.0 software.

## Descriptions

### Genus Dolabraulax

Quicke, 1986

Dolabraulax Quicke 1986: Ent. Mon. Mag. 122 (1): 18; Type species: Dolabraulax implicatusQuicke 1986.

#### General.

This genus may be separated from all other Braconinae by the combination of the following characters: scapus small, ventrally shorter than dorsally with dorso- and medio-basal expansions; propodeum posteriorly with a mid-longitudinal carina; and first metasomal tergite with the median area formed into a transverse ridge.

Dolabraulax is a small genus with only one species known from Java and the biology is unknown (Quicke, 1984). In this study, three new species of this genus are recognized, i.e. Dolabraulax jigongshanus sp. n., Dolabraulax brevivena sp. n. and Dolabraulax flavus sp. n., which are fully described and illustrated.

#### Key to the species of Dolabraulax Quicke

**Table:** 

1.	Propodeum with a completely mid-longitudinal carina or arising from the middle of the hind margin; vein 1-SR+M of fore wing slightly and evenly curved; smooth carina or band of second metasomal tergite relatively narrow baso-medially; fourth metasomal tergite with a transverse groove basally	2
–	Propodeum with an incompletely mid-longitudinal carina, arising from the 1/3 of the hind margin; vein 1-SR+M of fore wing straight; smooth carina or band of second metasomal tergite wide baso-medially ; fourth metasomal tergite without a transverse groove basally	3
2.	Frons weakly impressed behind each antennal socket, hardly divided by a raised, mid- longitudinal ridge; notauli impressed along its entire length (Fig. 1c); body largely yellowish brown; length of body 3.8 mm (central China)	Dolabraulax jigongshanus sp. n.
–	Frons deeply impressed behind each antennal socket, and distinctly divided by a raised, mid-longitudinal region; notauli only anteriorly impressed; body largely brown to black; length of body 4.9 mm (eastern Java)	Dolabraulax implicatus Quicke
3.	Face with long setae medially; propodeum with a longitudinal impressed area medially, densely setose laterally; vein r of fore wing short, 0.3 times as long as 2-SR (Fig. 3f); length of body 2.3 mm (eastern China)	Dolabraulax brevivena sp. n.
–	Face without long setae medially; propodeum without a longitudinal impressed area, but with a longitudinal carina medially, sparsely setose laterally (Fig. 2e); vein r of fore wing relatively long, 0.5 times as long as vein 2-SR (Fig. 2f); length of body 3.0–4.5 mm (south-western China)	Dolabraulax flavus sp. n.

#### Dolabraulax jigongshanus sp. n.

urn:lsid:zoobank.org:act:0C84C65B-4AE3-4AA3-9249-60F483729D64

Figs 1a–g 

##### Type specimens examined:

Holotype: ♀, Jigongshan, Henan, 12-VII-1997, Chen Xue-xin, No. 974960. Paratypes: 1♀1♂, Jigongshan, Henan, 12-VII-1997, Chen Xue-xin, No. 974894, 974881.

##### Description.

###### Length of body

3.8 mm, fore wing 3.4 mm, and ovipositor sheath 5.5 mm.

###### Head

**Figures 1a–g.**
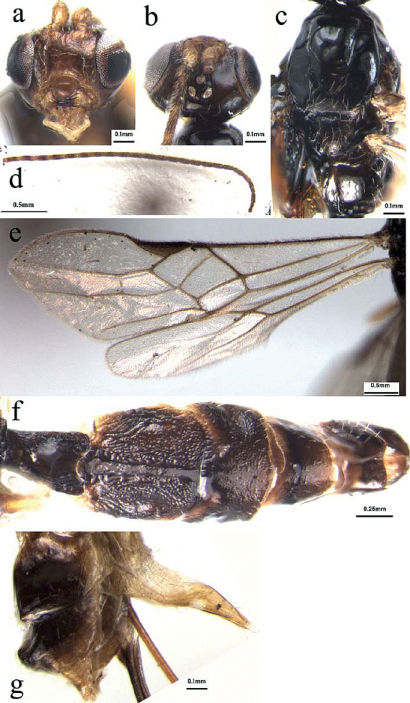
Dolabraulax jigongshanus sp. n.: **a** head, frontal view **b** head, dorsal view **c** mesoscutum, dorsal view **d** antenna **e** fore and hind wings **f** all of metasomal tergites, dorsal view **g** apical metasomal tergite, lateral view.

(Figs 1a–b, d): Antennae with 29 segments; first flagellomere parallel-sided, 3.1 times as long as its maximum width, 1.1 and 1.2 times as long as the second and third flagellomeres, respectively; second and third ones 2.8 and 2.9 times as long as their maximum width, respectively; median flagellomeres 2.6 times as long as their maximum width; terminal flagellomere tapering apically, approximately 2.9 times as long as its basal width; medio- transversal clypeal carina without a row sparse long setae; height of clypeus: inter-tentorial distance: tentorio-ocular distance = 1: 3: 5; malar space 0.35 times as long as height of eye; face with sparse long setae; height of face: width of face: width of head = 11: 13: 16; frons smooth and shiny, weakly impressed and without longitudinal ridge medially; shortest distance between posterior ocelli: diameter of posterior ocellus: shortest distance between posterior ocellus and eye = 2: 1: 7; vertex smooth and shiny, with sparse long setae medially.

###### Mesosoma

(Fig. 1c): Mesosoma 1.8 times as long as its maximum height, smooth and shiny with sparse long setae mid-posteriorly; notauli deeply impressed anteriorly and shallow posteriorly with sparse short setae along its whole length; middle lobe of mesoscutum rather raised anteriorly and protruding in front of the lateral lobes; scutellar sulcus relatively wide and deep, distinctly crenulate; metanotum with a strongly raised area medially; propodeum glabrous, with a mid-longitudinal carina and sparse setae, relatively densely setose laterally.

###### Wing

(Fig. 1e): Length of fore wing veins SR1: 3-SR: r = 38: 11: 7; vein 1-SR+M of fore wing weakly bent; length of fore wing veins 2-SR: 3-SR: r-m = 10: 11: 7; length of veins of hind wing SC+R1: 2-SC+R: 1r-m = 12: 4: 5; vein 2-SR+R of hind wing distinctly longitudinal; vein C+SC+R of hind wing with short thickened humeral bristles apically.

###### Leg:

Length of fore femur: tibia: tarsus = 23: 26: 34; length of hind femur: tibia: basitarsus = 25: 43: 9, and 3.9, 10.0 and 8.0 times their maximum width, respectively; tibia of hind leg with weakly longitudinal groove medially; spurs of hind leg 0.35 and 3.1 times as long as its basitarsus; tarsal claws simple but with basal lobe.

###### Metasoma

(Figs 1f, g): Metasoma distinctly longer than head and mesosoma combined, more or less parallel-sided; first tergite 1.3 times as long as its maximum apical width, with strongly raised area medio-apically and crenulate laterally, occupying four-fifths of its entire length; second tergite with medio-basal glabrous band reaching the suture between second and third tergites, and lateral depressed longitudinal grooves crenulate laterally, the remainder with rugulose sculpture; suture between second and third tergites deep and crenulate, wide medially and narrowed laterally; third tergite with distinct raised areas antero-laterally, smooth and shiny, with sparse setae apically; fourth-seventh tergites uniformly smooth and shiny, with sparse short setae apically; hypopygium acute apically, distinctly extending beyond apex of metasoma; ovipositor sheath 1.6 times as long as fore wing, with dense setae; ovipositor with teeth apico-ventrally and without dorsal notch pre-apically.

###### Colour:

Head dark yellow; antenna yellowish brown; face dark yellow; frons and vertex brown; mesosoma black; fore leg pale yellow, middle and hind legs dark yellow; pterostima yellowish brown; wings membrane pale grey, and veins dark yellow; metasomal tergites yellowish brown dorsally and pale yellow ventrally; ovipositor sheath yellowish brown.

###### Male:

Similar to the female, but relatively small, length of body 3.5 mm, metasoma black.

##### Biology:

Unknown.

##### Distribution:

China (Henan).

##### Etymology:

The new species is named after the type locality, Jigongshan in Henan (Central China).

##### Diagnosis:

This species is similar to Dolabraulax implicatus Quicke, but differs from the latter by the characters listed in the key above.

#### Dolabraulax flavus sp. n.

urn:lsid:zoobank.org:act:2AE73720-FF4D-4E89-91D0-215C8CDF352B

Figs 2a–h 

##### Type specimens examined:

Holotype: ♀, Guan Xian, Sichuan, 4-VIII-1980, He Jun-hua, No. 802020. Paratypes: 1♀, Guan Xian, Sichuan, 4-VIII-1980, He Jun-hua, No. 802020; 1♂, Emeishan, Sichuan, 7-VIII-1980, He Jun-hua, No. 802092; 1♀, Shaoguan, Guangdong, 12-V-1992, Chen Xue-Xin No. 921492; 1♀, Meifeng, Fujian, 27-VI-1962, Zhao Xiu-fu, No. 20004179.

##### Description.

###### Length of body

2.3 mm, fore wing 3.0 mm, and ovipositor sheath 4.1 mm.

###### Head

**Figures 2a–h.**
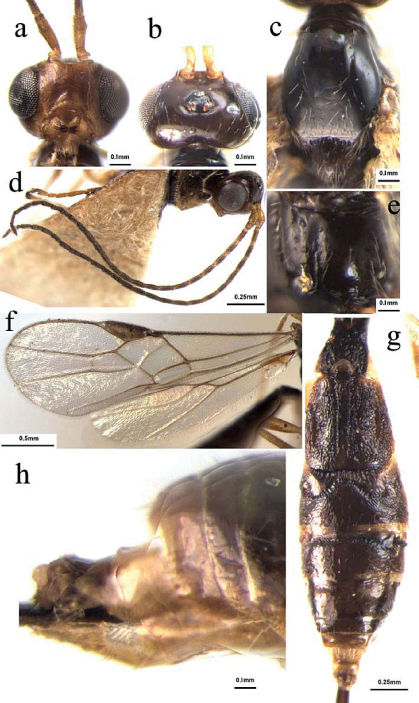
Dolabraulax flavus sp. n.: **a** head, frontal view **b** head, dorsal view **c** mesoscutum, dorsal view **d** antenna **e** propodeum, dorsal view **f** fore and hind wings **g** all of metasomal tergites, dorsal view **h** apical metasomal tergite, lateral view.

(Figs 2a–b, d): Antennae with 29 segments; first flagellomere parallel-sided, 2.9 times as long as its maximum width, 1.1 and 1.2 times as long as the second and third flagellomeres, respectively; second and third ones 2.7 and 2.8 times as long as their maximum width, respectively; median flagellomeres 2.6 times as long as their maximum width; terminal flagellomere tapering apically, approximately 3.2 times as long as its basal width; medio-transversal clypeal carina with a row sparse short setae; height of clypeus: inter-tentorial distance: tentorio-ocular distance = 9: 14: 22; malar space 0.34 times as long as height of eye; face with sparse long setae, relatively dense laterally; height of face: width of face: width of head = 12: 15: 17; frons smooth and shiny, weakly impressed, and without longitudinal ridge medially; shortest distance between posterior ocelli: diameter of posterior ocellus: shortest distance between posterior ocellus and eye = 2: 1: 7; vertex smooth and shiny, with sparse long setae medially.

###### Mesosoma

(Figs 2c, e): Mesosoma 1.6 times as long as its maximum height, smooth and shiny with sparse long setae mid-posteriorly; notauli deeply impressed half of the anterior and flat posteriorly with sparse long setae along its whole length; middle lobe of mesoscutum rather raised anteriorly and protruding in front of the lateral lobes; scutellar sulcus relatively wide and deep, with distinctly crenulate; metanotum with a strongly raised area medially; propodeum glabrous, with a mid-longitudinal impressed area and carina, arising from the 1/3 of the hind margin, with sparse setae, relatively densely setose laterally.

###### Wing

(Fig. 2f): Length of fore wing veins SR1: 3-SR: r = 40: 11: 6; vein 1-SR+M of fore wing weakly bent only medially; length of fore wing veins 2-SR: 3-SR: r-m = 10: 11: 7; length of veins of hind wing SC+R1: 2-SC+R: 1r-m = 12: 5: 4; vein 2-SR+R of hind wing distinctly longitudinal; vein C+SC+R of hind wing with short thickened humeral bristles apically.

###### Leg:

Length of fore femur: tibia: tarsus = 20: 24: 29; length of hind femur: tibia: basitarsus = 25: 38: 15, and 3.9, 10.5 and 7.0 times their maximum width, respectively; tibia of hind leg with weakly longitudinal groove medially; spurs of hind leg 0.34 and 3.0 times as long as its basitarsus; tarsal claws simple but with basal lobe.

###### Metasoma

(Fig. 2g, h): Metasoma distinctly longer than head and mesosoma combined, more or less parallel-sided; first tergite 1.4 times as long as its maximum apical width, with strongly raised area medio-apically, crenulate laterally, occupying three-fifths of its entire length; second tergite with medio-basal glabrous band hardly reaching the suture between second and third tergites, and lateral depressed longitudinal grooves crenulate laterally, the remainder with rugulose sculpture; suture between second and third tergites deep and crenulate, wide medially and narrow laterally; third tergite with distinct raised areas antero-laterally, smooth and shiny, with sparse setae apically; fourth tergite with transversal impressed groove basally; fourth-seventh tergites uniformly smooth and shiny, with sparse short setae apically; hypopygium acute apically, distinctly extending beyond apex of metasoma; ovipositor sheath 1.8 times as long as fore wing, with dense setae; ovipositor with teeth apico-ventrally and without dorsal notch pre-apically.

###### Colour:

Head dark yellow; antenna yellowish brown; face yellow; frons and vertex yellowish brown; mesosoma blackish brown; fore leg pale yellow, middle and hind legs dark yellow; pterostima yellowish brown; wings membrane pale grey, and veins dark yellow; metasomal tergites yellowish brown dorsally and pale yellow apically and ventrally; ovipositor sheath yellowish brown.

###### Male:

Similar to the female, but relatively small, length of body 2.2 mm, metasoma dark yellow.

##### Biology:

Unknown.

##### Distribution:

China (Fujian, Guangdong and Sichuan).

##### Etymology:

The new species is named after the colour of body, which is largely yellowish.

##### Diagnosis:

This species is similar to Dolabraulax implicatus Quicke, but differs from the latter by characters listed in the key above.

#### Dolabraulax brevivena sp. n.

urn:lsid:zoobank.org:act:7F7F9C17-B822-409E-8A71-2A973ECD58A0

Figs 3a–g 

##### Type specimens examined:

Holotype: ♀, Tianmushan, Zhejiang, 10–12, IX-1983, He Jun-hua, No. 83215. Paratypes: 3♀♀, Tianmushan, Zhejiang, 10–12, IX-1983, He Jun-hua, No. 832137, 832141, 832142; 1♂, west Tianmushan, Zhejiang, 16-V-1988, Lou Xiao-ming, No. 883232; 1♀, West Tianmushan, Zhejiang, 25-VI-1984, Zhu Xi-liang, No. 842055; 1♀, Longwangshan, Anji, Zhejiang, 31- VIII-1993, He Jun-hua, No. 9310586; 1♀, Si’an, Changxing, Zhejiang, 1-V-1984, Yuan Rong-lan, No. 940522.

##### Description.

###### Length of body

3.0–4.5 mm, fore wing 3.5–4.0 mm, and ovipositor sheath 5.0–5.5 mm.

###### Head

**Figures 3a–g.**
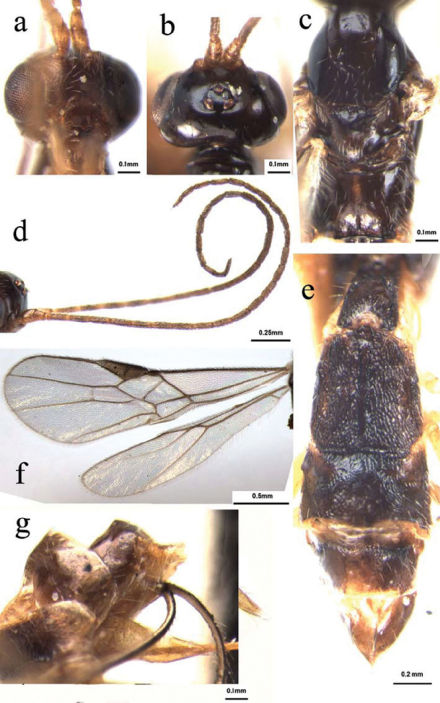
Dolabraulax brevivena sp. n.: a head, frontal view b head, dorsal view c mesoscutum, dorsal view d antenna e all of metasomal tergites, dorsal view f fore and hind wings g apical metasomal tergite, lateral view.

(Figs 3a–b, d): Antennae with 27 segments; first flagellomere parallel-sided, 3.0 times as long as its maximum width, 1.1 and 1.2 times as long as the second and third flagellomeres, respectively; second and third ones 2.5 and 2.7 times as long as their maximum width, respectively; median flagellomeres 2.6 times as long as their maximum width; terminal flagellomere tapering apically, approximately 3.4 times as long as its basal width; medio-transversal clypeal carina without a row sparse short setae; height of clypeus: inter-tentorial distance: tentorio-ocular distance =2.5: 6: 4; malar space 0.36 times as long as height of eye; face with sparse long setae, relatively dense laterally; height of face: width of face: width of head = 10: 11: 23; frons smooth and shiny, weakly impressed, without longitudinal ridge medially; shortest distance between posterior ocelli: diameter of posterior ocellus: shortest distance between posterior ocellus and eye = 2.5: 1: 7; vertex smooth and shiny, with sparse long setae laterally.

###### Mesosoma

(Fig. 3c): Mesosoma 1.8 times as long as its maximum height, smooth and shiny with dense long setae medio-posteriorly; notauli shallowly impressed half of the anterior and flat posteriorly with sparse long setae along its whole length; middle lobe of mesoscutum relatively raised anteriorlly and protruding in front of the lateral lobes; scutellar sulcus rather wide and deep, with distinctly crenulate; metanotum with a strongly raised area medially; propodeum glabrous, with a mid-longitudinal carina, arising from the 1/4 of the hind margin, with sparse setae medially, but relatively densely long setose laterally.

###### Wing

(Fig. 3f): vein r of fore wing relatively short, length of fore wing veins SR1: 3-SR: r = 37: 11: 6; vein 1-SR+M of fore wing weakly bent apically; length of fore wing veins 2-SR: 3-SR: r-m = 12: 11: 6; length of veins of hind wing SC+R1: 2-SC+R: 1r-m = 15: 3.5: 5; vein 2-SR+R of hind wing distinctly longitudinal; vein C+SC+R of hind wing with short thickened humeral bristles apically.

###### Leg:

Length of fore femur: tibia: tarsus = 21: 23: 29; length of hind femur: tibia: basitarsus = 12: 11: 6, and 4.1, 10.0 and 7.5 times their maximum width, respectively; tibia of hind leg with weakly longitudinal groove medially; spurs of hind leg 0.36 and 3.3 times as long as its basitarsus; tarsal claws simple but with basal lobe.

###### Metasoma

(Figs 3e, g): Metasoma distinctly longer than head and mesosoma combined, more or less parallel-sided; first tergite 1.3 times as long as its maximum apical width, with strongly raised area medio-apically, crenulate laterally, occupying four-fifths of its entire length; second tergite with medio-basal glabrous band hardly reaching the suture between second and third tergites, and lateral depressed longitudinal grooves crenulate laterally, the remainder with rugulose sculpture; suture between second and third tergites deep and crenulate, wide medially and relative narrow laterally; third tergite with distinct raised areas antero-laterally, smooth and shiny, with sparse setae apically; fourth tergite without transversal impressed groove basally; fourth-seventh tergites uniformly smooth and shiny, with sparse short setae apically; hypopygium acute apically, distinctly extending beyond apex of metasoma; ovipositor sheath 1.8 times as long as fore wing, with dense setae; ovipositor with teeth apico-ventrally and without dorsal notch pre-apically.

###### Colour:

Head reddish yellow; antenna dark yellow; face reddish yellow; frons and vertex yellowish brown; mesosoma dark brown; fore leg pale yellow, middle and hind legs dark yellow; pterostima yellowish brown; wings membrane pale grey, and veins dark yellow; metasomal tergites yellowish brown dorsally and pale yellow apically and ventrally; ovipositor sheath yellow brown.

###### Male:

Similar to the female, but relatively small, length of body 3.2 mm, metasoma dark yellow.

##### Biology:

Unknown.

##### Distribution:

China (Zhejiang).

##### Etymology:

The new species is named after the character of vein r of fore wing, which is relatively shorter.

##### Diagnosis:

This species is similar to Dolabraulax implicatus Quicke, but differs from the latter by characters listed in the key above.

### Genus Scutibracon

Quicke, 1989

Scutibracon Quicke 1989: Ento. Mon. Mag. 125: 19. Type species: Microbracon hispae Viereck 1913.

#### General.

This genus can be recognized by the following characters: small wasps with body length less than 3.0 mm;all flagellomeres more than twice times longer than wide; scapus small, shorter ventrally than dorsally in lateral view; face largely densely short-setose, smooth and shiny; frons distinctly impressed behind each antennal socket, short setose; scutellum densely and evenly setose; propodeum rather flat, with a complete mid-longitudinal carina; marginal cell of fore wing long, second submarginal cell of fore wing short, parallel-sided and robust, vein cu-a of fore wing distinctly postfurcal; claws with pointed basal lobes; first metasomal tergite with distinctly dorso-lateral carinae, second and third metasomal tergites enlarged, broad and short, and the third metasomal tergite more than 3.0 times wider than long medially. Species of this genus have been reared from Hispa armigera Olivier (Coleoptera: Hispidae) and Acrocercops cramerella Snellen (Lepidoptera: Gracilariidae).

Scutibracon is a small genus with only one known species from Indo-Australian (Quicke, 1984). In this study, one new species of this genus is added, Scutibracon fujianensis sp. n., which is described and illustrated below.

#### Scutibracon hispae

(Viereck, 1915)

Microbracon hispae Viereck 1915: Proc. U. S. Natn. Mus. 44: 639–648.
Bracon hispae : Watanabe 1937: J. Fac. Agric. Hokkaido Univ. 42: 1–188.
Scutibracon  hispae Quicke and Walker 1989: Ent. Mon. Mag. 125 (1): 19–20; He et al. 2002: Forest insects of Hainan, 883.

##### Biology:

According to the literature, it has been reared from Hispa armigera Olivier (Coleoptera: Hispidae), mostly on rice (Quicke 1989; He et al. 2002).

##### Distribution:

China (Hunan, Taiwan and Hainan), India and Java.

#### Scutibracon fujianensis sp. n.

urn:lsid:zoobank.org:act:56D83B91-84B3-479D-92B7-4F41304924F2

Figs 4a–i 

##### Type specimens examined

: Holotype: ♀, small Wuyishan, Fujian, 26–29-VII-1983, He Jun-hua, Ex. Acrocercops cramerella Snellen, No. 832849. Paratype: 1♀, Zhangzhou, Fujian, 9-X-1983, Wu Huang-quan, No. 881417.

##### Description.

###### Length of body

2.6 mm, fore wing 2.7 mm, and ovipositor sheath 0.7 mm.

###### Head

**Figures 4a–i.**
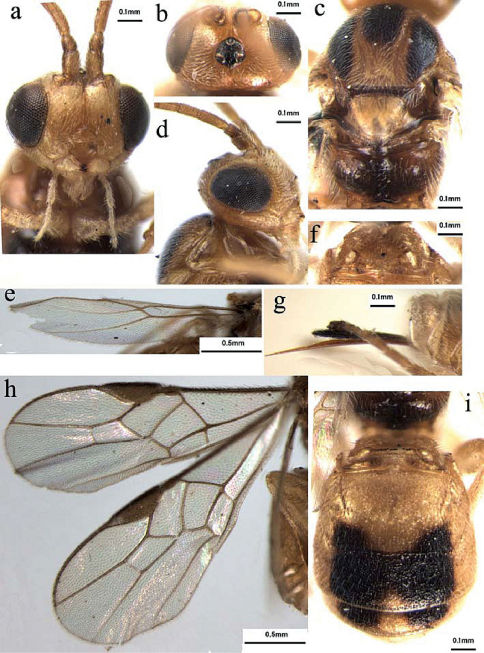
Scutibracon fujianensis sp. n.: **a** head, frontal view **b** head, dorsal view **c** mesoscutum and propodeum, dorsal view **d** head, lateral view **e** hind wing **f** first metasomal tergite, dorsal view **g** apical metasomal tergite, lateral view **h** fore wing **i** all of metasomal tergites, dorsal view.

(Figs 4a–b, d): Antennae as long as the fore wing, with 30 segments; scapus slightly flared apico-ventrally, distinctly weakly emarginated apico-laterally; first flagellomere parallel-sided, 1.5 times as long as the second flagellomeres; the latter 1.8 times as long as its maximum width; median flagellomeres 1.5 times as long as its maximum width; terminal flagellomere tapering apically, approximately 2.1 times as long as its basal width; medio-transversal clypeal carina with a row sparse short setae; height of clypeus: inter-tentorial distance: tentorio-ocular distance = 3: 6: 5; malar space 0.23 times as long as height of eyes; face with dense short setae, width of face: width of head: maximum length of eye in dorsal view = 15: 28: 14; frons smooth and shiny, densely short setose, strongly impressed and with longitudinal groove medially; shortest distance between posterior ocelli: diameter of posterior ocellus: shortest distance between posterior ocellus and eye = 2.5: 2: 6; vertex smooth and shiny, with dense setae.

###### Mesosoma

(Fig. 4c): Mesosoma 1.2 times as long as its maximum height, smooth and shiny, densely evenly short setae; notauli deeply impressed along its whole length; middle lobe of mesoscutum strongly raised anteriorlly; scutellar sulcus relatively wide and deep, with distinctly crenulate; metanotum with strongly raised area medially; propodeum glabrous, with a completely mid-longitudinal carina, and sparse setae medially, but relatively densely setose laterally.

###### Wing

(Figs 4e, h): Length of fore wing veins SR1: 3-SR: r = 27: 11: 7; vein 1-SR+M of fore wing weakly bent; length of fore wing veins 2-SR: 3-SR: r-m = 9: 11: 6; vein cu-a of fore wing distinctly postfurcal. Length of veins of hind wing SC+R1: 2-SC+R: 1r-m = 11: 2: 4; vein C+SC+R of hind wing without thickened humeral bristles apically.

###### Leg:

Length of fore femur: tibia: tarsus = 15: 19: 23; length of hind femur: tibia: basitarsus = 22: 28: 12, and 3.2, 4.7 and 4.2 times their maximum width, respectively; tibia of hind leg without longitudinal groove medially; spur of hind leg 0.36 and 3.2 times as long as its basitarsus; tarsal claws simple and without basal lobe.

###### Metasoma

(Figs 4f, g, i): Metasoma short and robust, evenly irregularly sculptured, slightly shorter than head and mesosoma combined; first metasomal tergite 1.3 times as wide as its maximum median long, with strongly raised area mid-apically occupying two-threes of its entire length, posterior part with coarse foveate rugose sculpture; second tergite without mid-basal triangular area, 2.0 times as long as its maximum width, with sub-lateral grooves medio-basally, extending to half of its entire length; third tergite broad and short, 2.7 times as wide as median long; suture between second and third tergites deep and crenulate, moderately wide; four tergite 0.5 times as long as third tergite medially; sixth-seventh tergites invisible, hypopygium short, acute apically, hardly extending beyond apex of metasoma; ovipositor sheath 0.25 times as long as fore wing, with dense setae; ovipositor without teeth apico-ventrally and dorsal notch pre-apically.

###### Colour:

Head yellow except for interocellar area black; mesosoma orange yellow but media and lateral lobes of mesoscutum with blackish spots; fore leg pale yellow, middle and hind legs dark yellow; wings membrane smokish grey, and veins yellowish brown; propodeum pale brown; metasomal tergites largely pale yellow but second metasomal tergite with blackish spots mid- apically, third metasomal tergite with black medially, four metasomal tergite with blackish spots sub-laterally; ovipositor sheath blackish brown.

##### Biology:

Based on labels of type specimens, the host of this species is Acrocercops cramerella Snellen (Lepidoptera: Gracilariidae).

##### Distribution:

China (Fujian).

##### Etymology:

The new species is named after the name of Fujian province, where the type specimens are collected.

##### Diagnosis:

This species is similar toScutibracon hispae (Viereck), but distinctly differs from the latter by having the vein r of fore wing longer, 0.6 times as long as vein 3-SR (Fig. 4h); the second tergite without a mid-basal triangular area, 2.0 times as long as its maximum width, with sublateral longitudinal grooves medio-basally, extending to half of its length (Fig. 4i); the interocellar area black (Fig. 4b); the middle and lateral lobes of mesoscutum with distinct blackish spots (Fig. 4c); the second- fourth tergites with blackish spots medio-apically, medially and sublaterally, respectively (Fig. 4i) and the length of body more than 3.0 mm.

## Supplementary Material

XML Treatment for Dolabraulax

XML Treatment for Scutibracon
